# A Transcriptomic Analysis of Human iPSC-Derived Parathyroid Lineage Cells Reveals Limited Maturation Beyond the Parathyroid–Thymic Primordium

**DOI:** 10.3390/cells15141252

**Published:** 2026-07-11

**Authors:** Chie Kise, Ryusuke Nakatsuka, Akiyo Kawamoto, Yuka Sasaki, Hirofumi Hitomi, Kazuya Takahashi, Tadashige Nozaki

**Affiliations:** 1 Department of Geriatric Dentistry, Faculty of Dentistry, Osaka Dental University, Hirakata 573-1121, Osaka, Japan; 2 Department of Pharmacology, Faculty of Dentistry, Osaka Dental University, Hirakata 573-1121, Osaka, Japan; 3 Department of iPS Stem Cell Regenerative Medicine, Faculty of Medicine, Kansai Medical University, Hirakata 573-1010, Osaka, Japan

**Keywords:** parathyroid differentiation, parathyroid–thymic primordium, human iPS cell, TBX1, gene expression profiling, transcriptome

## Abstract

**Highlights:**

**What are the main findings?**

- Human iPSC-based differentiation induces the parathyroid–thymic primordium stage.
- Differentiated cells are transcriptionally distinct from mature parathyroid and a developmental bottleneck limits parathyroid lineage commitment.

**What are the implications of the main findings?**

- Current human iPSC-based in vitro differentiation protocols induce parathyroid–thymic primordium differentiation through activation of pharyngeal pouch development-related genes.
- These protocols are insufficient to overcome the developmental bottleneck in parathyroid maturation.

**Abstract:**

Several protocols have been reported for inducing parathyroid differentiation from human pluripotent stem cells in vitro. However, the efficiency of terminal differentiation into mature parathyroid cells remains limited. In this study, we performed comparative transcriptomic analyses of parathyroid lineage-induced human induced pluripotent stem (iPS) cells to clarify their developmental stage identity. Comparative analyses with adult parathyroid adenoma, used as a surrogate reference for mature parathyroid cells, and thymuses revealed that the differentiated cells exhibited gene expression patterns consistent with early-stage parathyroid and thymus development, including TBX1, which is required for fate determination of the parathyroid–thymic primordium. On the other hand, the expression of mature parathyroid marker genes, such as *PTH*, *GCM2*, and the calcium-sensing receptor (*CaSR*), remained markedly lower than that in parathyroid adenoma. Under the differentiation conditions and durations examined, application of modified differentiation protocols did not significantly enhance the expression of mature parathyroid markers, although pharyngeal pouch developmental gene expression was maintained. Our findings indicate that current in vitro differentiation systems effectively induce the parathyroid–thymic primordium stage, while a developmental bottleneck at the transition from the primordium toward parathyroid lineage commitment remains.

## 1. Introduction

Attempts have been made to induce parathyroid cell differentiation from pluripotent stem cells, and several studies have described the successful differentiation of pluripotent stem cells into parathyroid hormone (PTH)-expressing cells [1,2,3]. The basic strategy for the directed differentiation of parathyroid cells involves stepwise induction through definitive endoderm (DE) and anterior foregut endoderm (AFE) intermediates [4,5,6,7,8]. In vivo organogenesis of parathyroid tissue originates from the pharyngeal pouch endoderm [9]. During pharyngeal pouch patterning and specification of the parathyroid primordium, differentiation-related transcription factors and their signaling pathways, including TBX1, EYA1, HOXA3, GATA3, and FOXN1, are orchestrated [10,11,12,13]. These factors induce endodermal tissue toward the parathyroid lineage through spatially and temporally restricted expression. In parathyroid lineage commitment and maturation, glial cell missing 2 (GCM2) plays a critical role in parathyroid development because its loss of function causes congenital hypoparathyroidism [14,15,16].

Using development-based parathyroid cell induction methods, Bingham et al. first reported the expression of PTH and the calcium-sensing receptor (CaSR) [17]. Recent studies on the in vitro differentiation of parathyroid cells have demonstrated the expression of parathyroid markers. However, the maturation of parathyroid lineage-differentiated cells and their physiological functions, such as responsiveness to extracellular calcium, remain inconsistent [1,2,3]. Various procedures have been developed to improve the efficiency of differentiation induction. Regarding priming toward the DE and AFE stages, it has been reported that retinoic acid, Wnt, and BMP signaling pathways play essential roles in AFE patterning of DE in pluripotent stem cells [7]. CDK4/6 inhibitors have also been reported to enhance the efficiency of endoderm specification during early endoderm differentiation in parathyroid differentiation induction [18]. Parathyroid organoids derived from induced pluripotent stem cells (iPSCs) have exhibited calcium-responsive PTH secretion and morphological characteristics resembling those of the parathyroid gland [2]. Furthermore, our previous report indicated that TGF-α/EGFR signaling promotes parathyroid cell differentiation from iPSCs. Despite various improvements in differentiation efficiency, the generation of physiologically mature parathyroid cells remains unresolved.

During organogenesis, various signaling pathways are utilized throughout development, and cells respond in a stage-dependent manner. Transcriptomic transitions are associated with differentiation stage-specific cellular states. Transcriptome profiling of endoderm populations has revealed the transcriptional characteristics of the ontogeny of the endodermal organ system, including parathyroid gland development [19]. These findings imply that the transcriptional patterning of parathyroid lineage-differentiated cells is related to transitional states and lineage commitment during thymus and parathyroid organogenesis. Therefore, understanding the transcriptome profiles during parathyroid development will provide insight into the differentiation stage-specific cellular states. Several studies on in vitro parathyroid differentiation have elucidated differentiation efficiency based on the expression of selected marker genes and functional assays [1,2,3]. However, a comprehensive analysis of the gene expression profiles between differentiated cells and tissue-derived parathyroid glands has not been performed from a developmental perspective, and the developmental stage identity of differentiated cells has not been defined. In the current protocols of differentiation induction, it remains uncertain whether differentiated cells reach a mature parathyroid stage or remain at the parathyroid–thymic primordium stage. Therefore, elucidating the embryological characteristics of parathyroid lineage-differentiated cells is important for understanding the developmental bottlenecks that may restrict maturation toward fully functional parathyroid cells.

In the present study, we performed comprehensive transcriptomic comparisons among human iPSC-derived parathyroid lineage-differentiated cells, parathyroid adenomas that retain the characteristics of mature parathyroid cells, and the thymus to determine their developmental stage identity. We aimed to determine whether established differentiation procedures induce lineage commitment to the parathyroid–thymic primordium stage or the mature parathyroid stage. Furthermore, we investigated the effects of modified differentiation protocols on the expression of parathyroid maturation-associated genes. Our findings provide valuable insight into the developmental characteristics of parathyroid lineage differentiation and offer a basis for improving differentiation efficiency.

## 2. Materials and Methods

### 2.1. Preparation and Maintenance of Human iPSCs

Experiments using human induced pluripotent stem cells (hiPSCs) were approved by the Ethics Committee of Kansai Medical University and Osaka Dental University. Informed consent was obtained from the donor from whom the hiPSCs were derived in accordance with the guidelines of the Declaration of Helsinki. All methods were performed in accordance with the institutional guidelines. A human iPSC line of iPSC #5 cells was generated from peripheral blood-derived mononuclear cells and maintained in xeno-free media (StemFit AK02N medium; Ajinomoto, Co., Ltd., Tokyo, Japan) on iMatrix-511 silk (Matrixome, Osaka, Japan)-coated cell culture dishes for experiments up to passage 20. In brief, cells were passaged using cell dissociation reagent (Accutase; Nacalai Tesque, Kyoto, Japan) supplemented with 10 µM Y-27632 (Toronto Research Chemicals Inc., North York, ON, Canada) and seeded onto an iMatrix-511 silk-coated 60 mm culture dish in StemFit AK02N medium supplemented with 10 µM Y-27632. The medium was replaced with StemFit AK02N medium without Y-27632 one day after passaging. Cells were manipulated in a clean bench under germ-free conditions and tested negative for mycoplasma by staining with Hoechst 33258.

### 2.2. Parathyroid Differentiation of Human iPSCs

The differentiation protocol has been described previously [1]. For differentiation, human iPSCs were dissociated with accutase (Nacalai Tesque) and replated onto matrigel (Corning Inc., Corning, NY, USA)-coated 12-well cell culture plates at a density of 50,000 cells/cm^2^. The next day, the cells were cultured in RPMI 1640 (Nacalai Tesque) supplemented with 2% B-27 supplement (Thermo Fisher Scientific, Waltham, MA, USA) containing 3 μM CHIR-99021 (Focus Biomolecules, Plymouth Meeting, PA, USA) and 100 ng/mL activin A (API Co., Ltd., Gifu, Japan). The cells were cultured with differentiation medium (RPMI 1640 supplemented with 2% B-27 supplement) containing 100 ng/mL activin A and 500 nM LDN 193189 (Merck, Darmstadt, Germany) for another 2 days. The cells were then cultured with differentiation medium for three days. In stage 2, the differentiated cells were cultured with 1 μM ATRA (LKT Laboratories, Saint Paul, MN, USA) and 2.5 μM IWR1-endo (Fujifilm, Tokyo, Japan) for 4 days. Finally, in stage 3, we treated the cells according to several protocols for the induction of parathyroid cells; in our original protocol [1], the cells were treated with 100 ng/mL SHH (PeproTech, Cranbury, NJ, USA) and 50 ng/mL activin A (Protocol-1). Two other modified protocols were originated from previously reported studies [2,3]. The first protocol: The cells were cultured in DMEM/Ham’s F-12 medium (DMEM/F-12; Nacalai Tesque) supplemented with 2% serum replacement regent (StemSure Serum Replacement; SSR, Fujifilm) containing 1 μM Alk-5 inhibitor (A83-01; Stemgent, Cambridge, MA, USA), 100 nM ATRA, 50 ng/mL FGF10 (Miltenyi Biotec, Bergisch Gladbach, Germany), and 1 ng/mL BMP4 (PeproTech) for another 2 days, and then the cells were cultured in RPMI 1640 supplemented with 2% SSR containing 1 µM A83-01, 100 nM ATRA, 50 ng/mL FGF10, 400 ng/mL Noggin (Miltenyi Biotec), and 10 nM dexamethasone (Fujifilm) (Protocol-2). The other protocol: The cells were treated with 1 µM A83-01, 100 nM ATRA, 100 ng/mL SHH, and 10 ng/mL BMP4 for another 2 days, and then cells were cultured with 1 μM A83-01, 100 nM ATRA, 100 ng/mL SHH, and 200 ng/mL Noggin (Protocol-3).

### 2.3. Total RNA Preparation and the RNA Sequencing Analysis of Total RNAs

After 15 days of differentiation induction, total RNA from differentiated cells was isolated using the RNeasy Plus Mini kit (Qiagen, Venlo, The Netherlands) according to the manufacturer’s instructions. We prepared the derived total RNAs of differentiated cells, adenoma of the parathyroid gland (CR559547; Origene, Rockville, MD, USA), and thymus cells (QS0630; Thermo Fisher Scientific). Total RNA from normal human parathyroid is difficult to obtain due to ethical and practical limitations. Therefore, parathyroid adenoma samples were used as a surrogate for normal parathyroid. Parathyroid adenomas retain functional characteristics of mature parathyroid, including PTH expression and responsiveness to extracellular calcium. The concentration and purity of the collected RNA were evaluated using a spectrophotometer (Nanodrop; Thermo Fisher Scientific). RNA sequencing (RNA-seq) was performed using a NovaSeq X Plus (Illumina San Diego, CA, USA) sequencer, and all procedures for library preparation and sequencing analysis were performed by Rhelixa Co., Ltd. (Tokyo, Japan). The total RNA samples were converted to cDNAs with oligo dT primers using a NEBNext Poly (A) Magnetic Isolation Module (for poly A selection; New England Biolabs, Frankfurt, Germany) and a NEB Next Directional Ultra II RNA Library Prep Kit (for strand-specific libraries; New England Biolabs). These cDNA libraries were sequenced with a NovaSeq X Plus to produce 150 base pair-end reads. The nucleotide sequence data have been deposited at the DDBJ Sequenced Read Archive under BioProject accession number PRJDB40433 and are publicly available as of the date of publication.

### 2.4. Gene Set Enrichment Analysis

A comparison analysis of the RNA-seq data was performed using a gene set enrichment analysis (GSEA) software program (https://www.gsea-msigdb.org/gsea/index.jsp; accessed on 28 January 2025) in accordance with previous reports [20]. For the GSEA, cell type signature gene sets (C8) were utilized, and the enrichment plots were visualized.

### 2.5. Immunofluorescence Staining

The iPSCs were replated onto Matrigel-coated 2-well cell culture slides (Corning) at a density of 50,000 cells/cm^2^ and differentiated into the parathyroid lineage. After 15 days of differentiation induction, the differentiated cells were washed twice with PBS^−^ and fixed with 4% paraformaldehyde. The fixed cells were permeabilized with 0.1% Triton X-100 and pre-incubated with PBS^−^ containing 10% chicken serum to reduce nonspecific binding. After blocking, the cells were incubated overnight at 4 °C in a humidified chamber with the primary antibody against TBX1 (Thermo Fisher Scientific; 1:25 dilution), EYA1 (Proteintech, Rosemont, IL, USA; 1:200 dilution) and PTH (R&D Systems, Minneapolis, MN, USA; 1:100 dilution). Subsequently, the cells were incubated with Alexa Fluor-594-conjugated and Alexa Fluor-488-conjugated secondary antibodies (Thermo Fisher Scientific; 1:500 dilution). The nuclei were counterstained with Hoechst 33342 (Thermo Fisher Scientific), and the cells were observed using a BZ-X800 fluorescence microscope (Keyence, Osaka, Japan). For the quantitative evaluation of the TBX1 positive cell proportion, the mean intensity of TBX1 and nuclei fluorescence of differentiated cells in the eleven pictured fields were analyzed, and the percentage of TBX1 positive cells was quantified by ImageJ software (ver. 1.53e; https://imagej.net/ij/; 16 September 2020).

### 2.6. Statistical Analyses

After normalization of the dataset, Pearson’s correlation coefficients (r) of the normalized counts were calculated to assess the correlation between samples. For comparisons among differentiation protocols (Protocols 1–3), data were evaluated by a one-way ANOVA with Tukey’s multiple comparison procedure. For transcriptomic comparisons between differentiated cells and reference samples (parathyroid adenoma and thymus), statistical analyses were not performed because the reference datasets were derived from single RNA samples (n = 1). Statistical significance was defined as follows: * *p* < 0.05.

## 3. Results

### 3.1. A Comprehensive Gene Expression Analysis of Parathyroid-like Differentiated Cells, Parathyroid Adenoma and Thymus

**Figure 1:**
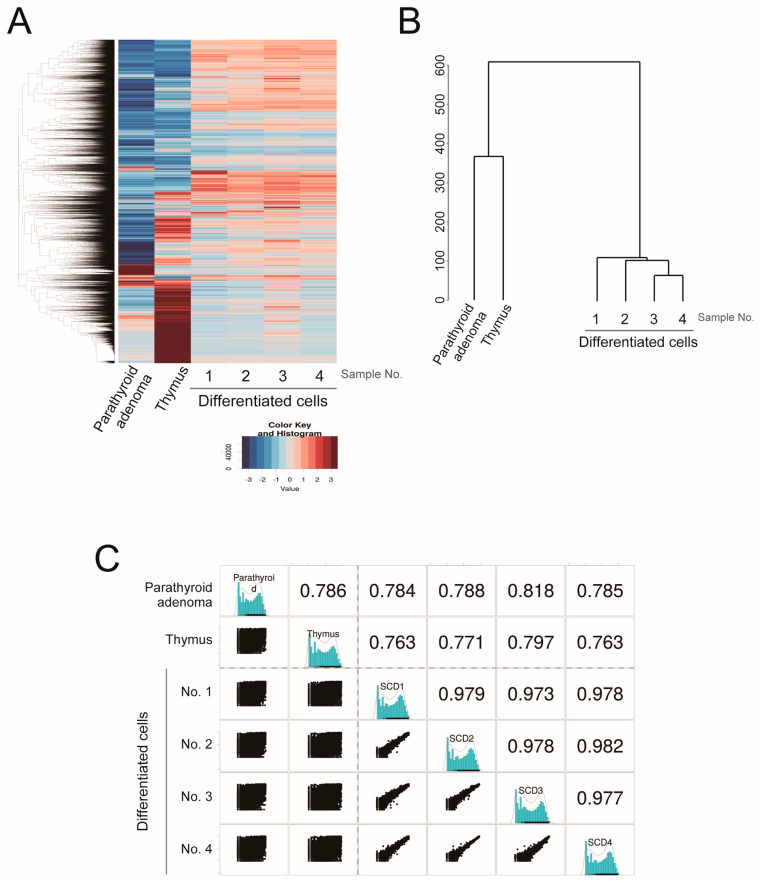
**Comparison of the gene expression profiles among the iPSC-derived parathyroid lineage-differentiated cells, parathyroid adenomas, and thymus cells.** (**A**) RNA-seq data from parathyroid adenomas, thymus cells, and parathyroid lineage-induced human iPSCs (four independent samples, nos. 1 to 4) were normalized using the transcripts per million (TPM) method and visualized as a heat map. Individual Z-scores are shown in color according to the indicated color key. (**B**) A hierarchical clustering dendrogram of differentially expressed genes across the three experimental groups. The gene expression values were normalized and scaled prior to clustering. The vertical axis indicates the distance between clusters. (**C**) Pearson correlation coefficients of the gene expression profiles are presented in a matrix format. The calculated correlation coefficient (r) is shown in each cell of the matrix.

To examine whether parathyroid lineage-induced human iPSCs show gene expression similarity with parathyroid or thymic tissues, comprehensive gene expression analyses were performed comparing cells differentiated using our established protocol with parathyroid adenoma or thymus cells. As shown in the hierarchical clustering analysis (Figure 1), the differentiated cells (sample nos. 1–4) did not form distinct clusters with either parathyroid adenoma or thymus cells (Figure 1A). In contrast, each differentiated cell sample showed similar gene expression clustering. These differences in transcriptional profiles were confirmed using a dendrogram analysis (Figure 1B). The differentiated cells were characterized by separate branches from parathyroid adenoma and thymus cells. A pairwise correlation analysis also indicated low genetic relatedness between differentiated cells and parathyroid adenoma or thymus cells (Figure 1C). While strong correlations were observed among differentiated cell samples (r = 0.973–0.982), correlations between differentiated cells and parathyroid adenoma or thymus cells were moderate (parathyroid adenoma, r = 0.784–0.818; thymus, r = 0.763–0.797). These results suggest that differentiated cells are transcriptionally distinct from mature parathyroid tissue and thymus cells.

### 3.2. A Transcriptomic Analysis of Developmental Related Genes Toward Parathyroid and Thymic Lineages

**Figure 2:**
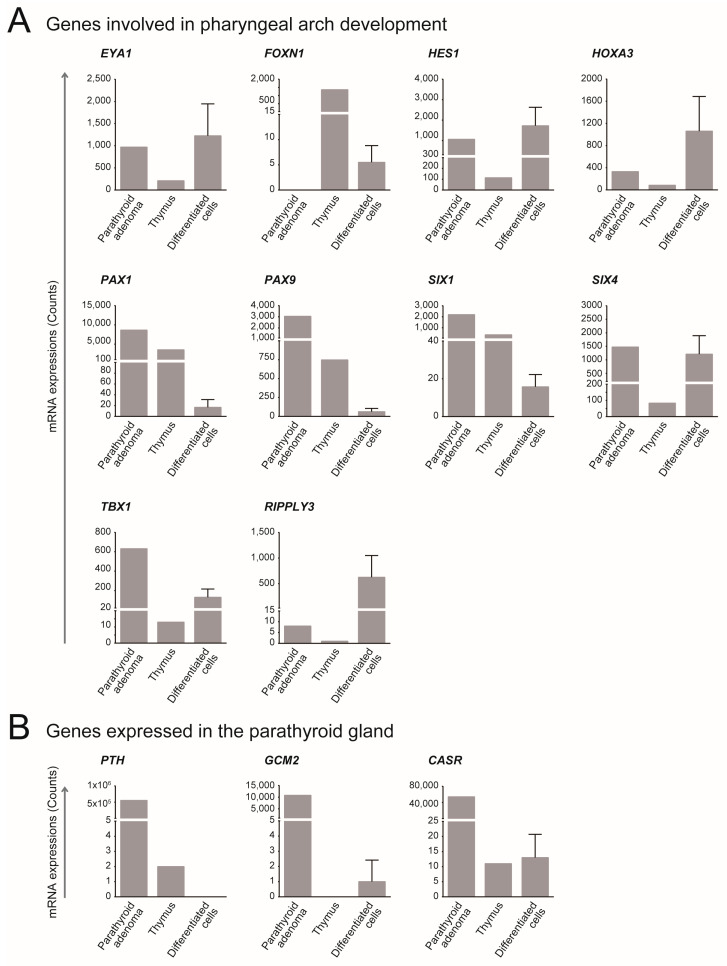
**Gene expression profiling during parathyroid and thymic primordium development.** Normalized gene counts derived from RNA-seq data are shown. (**A**) mRNA expression of pharyngeal arch stage-specific genes. (**B**) The mRNA expression of mature parathyroid gland-specific genes. Bars represent the mean ± SD of four independent samples of parathyroid lineage-induced human iPSCs. Comparisons are presented descriptively, and no statistical analyses were performed.

Although the gene expression patterns of differentiated cells are thought to be distinct from those of mature parathyroid tissue and thymus cells (Figure 1), the currently used parathyroid cell induction method is based, in part, on embryological development. Therefore, we investigated the expression of pharyngeal arch stage-specific genes involved in parathyroid–thymic primordium development (Figure 2). As shown in Figure 2A, the genes involved in early pharyngeal arch patterning and primordium formation, such as *EYA1*, *HES1*, *HOXA3*, and *TBX1*, were expressed in differentiated cells. In particular, *RIPPLY3* was expressed at higher levels than in parathyroid adenoma and thymus cells. In contrast, the expression levels of *PAX1*, *PAX9*, and *SIX1*, which are transcription factors implicated in later stages of pharyngeal pouch specification, decreased (Figure 2A). Furthermore, the expression of marker genes of mature parathyroid cells, such as *PTH*, *GCM2*, and the calcium-sensing receptor (*CaSR*), remained insufficient compared to that in parathyroid adenoma and thymus cells (Figure 2B). These results suggest that differentiation into the early pharyngeal arch stage, which functions as the parathyroid–thymic primordium, was effectively induced, whereas commitment to physiological parathyroid cells was insufficient when compared with parathyroid adenoma under the current differentiation duration and conditions.

### 3.3. TBX1 Expression in Parathyroid-like Cell Differentiation Cultures

**Figure 3:**
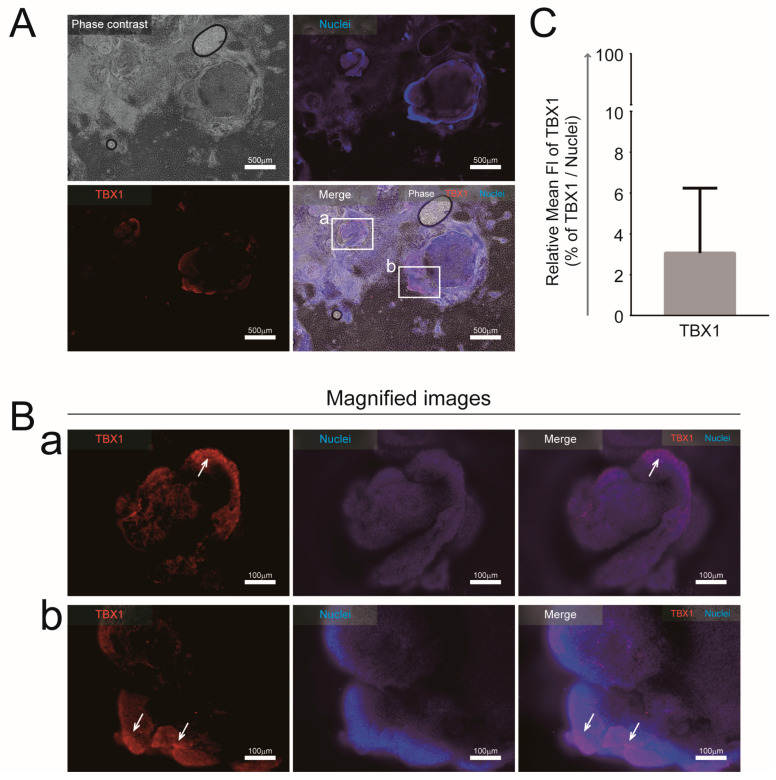
**The expression and localization of TBX1 in the iPSC-derived parathyroid lineage-differentiated cells.** (**A**) A representative phase contrast image and fluorescence images of differentiated cells in culture (scale bars: 500 μm). The fluorescence images of TBX1 (red) and nuclei (blue) in parathyroid differentiated cells are shown. (**B**) Magnified images of parathyroid differentiated cells (scale bars: 100 μm). The area indicated by white squares in low-magnification images (**a**,**b**) is magnified. The peripheral expression of TBX1 is indicated with arrows. (**C**) The percentage of TBX1-positive cells was quantified. TBX1 and nuclear fluorescence in differentiated cells were analyzed by fluorescence microscopy. Quantification was performed based on the MFI obtained from eleven independent pictured fields. Bars represent the mean ± SD of the calculated value.

As indicated in Figure 2A, *TBX1*, a regulatory factor for parathyroid–thymic primordium development, was expressed in differentiated cells. Therefore, we investigated the protein expression and localization of TBX1 in cultured cells (Figure 3). As shown in Figure 3A, TBX1 localized to cell aggregates and it localized to the nuclei. Meanwhile, its expression was not ubiquitous. These expression patterns were confirmed using high-magnification images; that is, the TBX1 expression was restricted to the marginal regions of the cell aggregates (Figure 3B). Regarding another marker of early parathyroid development, EYA1, which was highly expressed in differentiated cells (Figure 2A), exhibited an expression pattern similar to that of TBX1 (Figure S1A). In addition, the proportion of TBX1 positive cells appeared relatively low (Figure 3C). Among the TBX1-expressing cells, only a few cell aggregates expressed PTH, whereas the majority of cell aggregates lacked detectable PTH expression (Figure S1B). These results indicate that the TBX1 expression was not homogeneous in parathyroid lineage-induced cells, and a subset of cells differentiated into the parathyroid–thymic primordium, whereas others remained poorly differentiated.

### 3.4. Comparison of Culture Conditions for Parathyroid Cell Differentiation

To determine whether the efficiency of parathyroid lineage differentiation differs depending on the culture method, we compared a 3D spheroid differentiation model with a 2D planar differentiation model. In the 2D planar differentiation model, scattered cell aggregates were observed throughout the culture, whereas larger spheroids formed under 3D culture conditions (Figure S2A). When the differentiation induction was prolonged, the expression levels of TBX1 and PTH were upregulated in both cultures (Figure S2B). However, their expression levels were comparable between the 2D and 3D cultures. In contrast, HOXA3 expression was higher in the 2D culture than in the 3D culture (Figure S2B).

### 3.5. Comparison of the Differentiation Protocols for Parathyroid Cell Maturation

**Figure 4:**
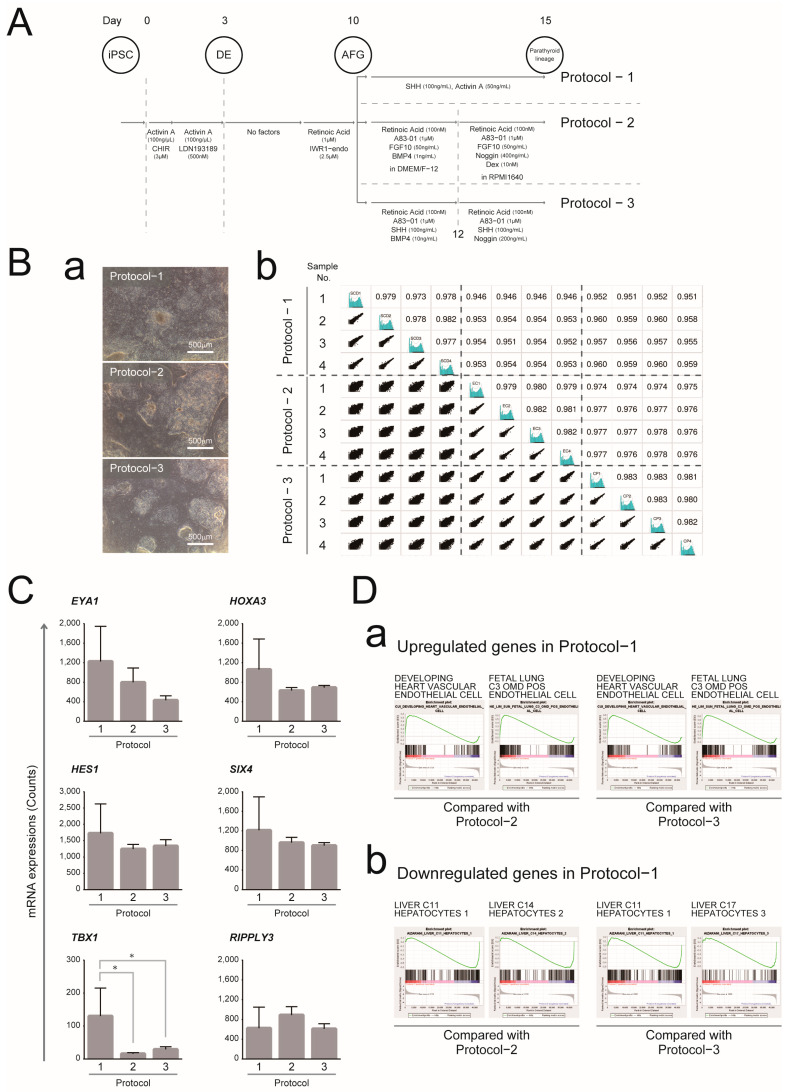
**Effects of the modified differentiation protocols on parathyroid cell differentiation and maturation.** (**A**) A schematic diagram of the modified differentiation protocol. Each differentiation stage during the 15-day differentiation period is shown in the circles (iPSC: induced pluripotent stem cells, DE: definitive endoderm, AFG: anterior foregut, and parathyroid lineage). After the 10th day of differentiation induction (AFG stage), these cells were subjected to three differentiation protocols indicated as Protocol-1, Protocol-2, and Protocol-3. The growth factors and chemical compounds are shown at the bottom of each panel. (**B**) (**a**) Representative phase-contrast images of parathyroid lineage-differentiated cells obtained using the three modified differentiation protocols at day 15 (scale bars: 500 μm). (**B**) (**b**) Pearson correlation coefficients of the gene expression profiles between the protocols (individual protocol performed in four independent samples, nos. 1 to 4) are presented in a matrix format. The calculated correlation coefficient (r) is shown in each cell of the matrix. (**C**) Comparison of the mRNA expression among the protocols. Normalized gene counts derived from the RNA-seq data are shown. The gene counts of these samples were compared; bars represent the mean ± SD of four independent measurements. The data were evaluated by a one-way ANOVA with Tukey’s multiple comparison procedure. Statistical significance was defined as follows: * *p* < 0.05. (**D**) GSEA of RNA-seq data comparing the original protocol (Protocol-1) with the modified protocols (Protocol-2 and 3). Among the early developmental patterning-related gene sets categorized in cell type signature gene sets (C8), (**a**) upregulated and (**b**) downregulated pathways are shown.

To investigate whether maturation into parathyroid cells under in vitro differentiation could be improved, we applied modified differentiation protocols referenced from previous studies. As shown in Figure 4A, our original protocol (Protocol 1) and the modified protocols based on previous studies (Protocols 2 and 3) were applied through the anterior foregut stage of differentiation (Day 10). In Protocol 2, the culture medium was changed to DMEM/F-12 during differentiation (Days 10–12; Figure 4A). Previous studies have shown that switching to DMEM/F-12 during the AFE stage improves PTH expression [3]. In addition, the modified protocols (Protocols 2 and 3) were refined to mimic the signaling environment associated with parathyroid development. For instance, BMP4 signaling contributes to parathyroid–thymic primordium formation but has been reported to inhibit parathyroid maturation. Accordingly, NOGGIN, a BMP antagonist, was administered after BMP4 stimulation in Protocols 2 and 3 to facilitate parathyroid maturation. Under all differentiation conditions, similar cell aggregations, presumed to represent parathyroid–thymic primordia, were formed by differentiation induction (Figure 4B(a)). Furthermore, the biological relatedness among these differentiated cells was indicated by a pairwise correlation analysis (Figure 4B(b)). We then analyzed the gene expression of pharyngeal arch patterning- and primordium formation-related genes. As a result, the expression levels of *EYA1*, *HES1*, *HOXA3*, and *SIX4* were comparable, although they showed little variation among the protocols (Figure 4C). In contrast, the *TBX1* expression in Protocol 1 was significantly higher than that in the other protocols, whereas *RIPPLY3*, which is located downstream of TBX1 during pharyngeal arch patterning, showed little change in expression (Figure 4C). We then interpreted the biological differences between Protocol 1 and the modified protocols (Protocols 2 and 3) using a gene set enrichment analysis (Figure 4D). In our established protocol, developing endothelial cell-related signatures were enriched (Figure 4D(a)), whereas hepatic lineage-related signatures were reduced compared with the modified protocols (Figure 4D(b)). Collectively, these results indicate that current methods for differentiation into parathyroid cells induce the parathyroid–thymic primordium with respect to the expression of pharyngeal arch patterning and primordium formation genes, such as *TBX1*. However, none of the differentiation protocols investigated in this study enhanced differentiation into physiological parathyroid cells.

## 4. Discussion

Several groups, including us, have reported the successful induction of parathyroid cell differentiation from human pluripotent stem cells. Early studies demonstrated the expression of parathyroid marker genes. However, it has been suggested that the maturation efficiency of the parathyroid lineage is limited [17]. Subsequently, several in vitro differentiation protocols were reported, including efficient methods for inducing parathyroid hormone-expressing cells from ES and iPSCs [1,2,3]. More recently, parathyroid organoids generated from iPSCs have been reported to enhance parathyroid cell function, including calcium-responsive PTH secretion, which corresponded with our results (Figure S2) [21]. While these studies have achieved the induction of several parathyroid marker genes, the efficiency, stability, and functional properties of the differentiated cells have been inconsistent. In particular, differences in maturity and the cellular function between pluripotent stem cell-derived parathyroid-like cells and native parathyroid cells remain unresolved. The use of parathyroid adenoma instead of normal parathyroid represents a limitation of this study. Normal human parathyroid tissue is difficult to obtain due to ethical and practical constraints. Parathyroid adenomas retain functional characteristics of differentiated parathyroid cells, including PTH expression and calcium-sensing machinery, even though they show lower sensitivity in extracellular calcium-PTH release than parathyroid glands [22]. Therefore, parathyroid adenoma is used as a surrogate model for differentiated parathyroid cells.

In the present study, we analyzed the comprehensive gene expression profiles of human iPSC-derived parathyroid lineage-differentiated cells. Comparative analyses of differentiated cells, parathyroid adenoma, and thymus cells revealed that differentiation into mature parathyroid cells is limited, whereas differentiation into the parathyroid–thymic primordium stage is effectively induced (Figure 1 and Figure 2). The genes related to pharyngeal pouch development, including *TBX1*, *HOXA3*, *EYA1*, and *HES1*, were markedly expressed in differentiated cells (Figure 2A). These genes are key regulators of parathyroid–thymic primordium development [13,23,24]. Among these genes, *RIPPLY3* was expressed at higher levels than in parathyroid adenoma or thymus (Figure 2A). *RIPPLY3* is regulated by retinoic acid signaling [25,26]. Because retinoic acid was included in all protocols (Protocols 1 to 3) during the pharyngeal pouch development and parathyroid lineage induction stages, *RIPPLY3* expression was observed across all protocols (Figure 4C). On the other hand, *TBX1* is an indispensable factor for parathyroid–thymic primordium development, and its deletion induces hypoplasia or agenesis of the parathyroid glands and thymus [10,27,28]. As shown in Figure 3, TBX1 was expressed within cell aggregates, which is consistent with parathyroid development. Notably, its expression was heterogeneous and restricted to the marginal regions of the aggregates rather than being uniformly distributed throughout the culture (Figure 3B). These findings suggest that only a subset of cells is committed to the parathyroid–thymic primordium, whereas others remain immature and/or committed to alternative endodermal lineages. Such restricted localization of the TBX1 expression is consistent with its tightly regulated expression during in vivo pharyngeal pouch morphogenesis.

Despite the induction of the regulatory genes involved in parathyroid–thymic primordium development, the expression levels of mature parathyroid cell markers, including *PTH*, *GCM2*, and *CaSR*, were markedly lower than those observed in parathyroid adenomas (Figure 2B). Whereas *PTH* expression was upregulated in the extended differentiation induction, PTH-positive cells were limited (Figures S1 and S2) [1]. Among these genes, *GCM2* plays a critical role in parathyroid lineage commitment and maintenance [14,16]. In rodent studies, the ablation of *Gcm2* results in the absence of parathyroid glands, and in humans, pathogenic variants in *GCM2* cause hypoparathyroidism [15,29]. Our recent study provided insight into the developmental role of GCM2 in parathyroid differentiation from iPSCs. Using a Tet-On inducible system, we demonstrated that GCM2 functions as a restrictive factor at premature differentiation stages, whereas at the pharyngeal pouch development stage, it enhances the expression of parathyroid markers [14]. These findings indicate that the temporal regulation of GCM2 is critical for parathyroid lineage commitment. In accordance with this phenomenon, the limited expression of *GCM2* observed in the present study suggests that current differentiation protocols are inefficient in promoting maturation beyond the parathyroid–thymic primordium stage. This interpretation is further supported by the finding that mature parathyroid cell markers were not upregulated even after the application of modified differentiation protocols referenced from preceding studies. However, the present study did not include an extended maturation phase applied in some previous reports [1,2,3,17]. Our previous study indicated that the proportion of presumed parathyroid-differentiated cells transiently increased up to day 17 and gradually decreased thereafter, while the expression levels of GCM2 and PTH remained unchanged [1]. Therefore, the observed transcriptional immaturity may reflect both limitations in the transition toward parathyroid lineage commitment and the limited maturation period. Although differences among differentiation protocols were observed in early developmental genes, such as *TBX1* (Figure 4C,D), these differences had little impact on the overall gene expression profiles and cellular morphology (Figure 4B). These results suggest that the optimization of differentiation steps through the anterior foregut stage alone may be insufficient to overcome the developmental bottleneck in parathyroid maturation.

In the present study, the differentiation protocols described in previous reports were modified to fit our experimental conditions. The composition of the supplemented factors and the duration of differentiation induction were adjusted for our experiments. These modifications were not intended to replicate the original protocols, but rather to focus on anterior foregut-to-pharyngeal pouch development. Neither extensive changes in gene expression nor the upregulation of mature parathyroid markers were observed despite the modifications applied to the differentiation protocols (Figure 4B,C). These findings suggest that differences in anterior foregut patterning alone may be insufficient to promote full maturation into physiological parathyroid cells.

During in vivo parathyroid organogenesis, differentiated cells are influenced by surrounding tissues, including the endodermal epithelium, mesenchyme, and neural crest-derived cells, as well as by gradients of signaling pathways, such as BMP, Wnt, and Sonic hedgehog [2]. In contrast, the current in vitro differentiation systems cannot fully reproduce the surrounding microenvironment, which likely contributes to the incomplete maturation observed in parathyroid differentiation in vitro. Taken together, the results of the present study suggest that a major limitation under the conditions examined in this study lies in the commitment and maturation processes toward the mature parathyroid lineage, rather than in the differentiation into the parathyroid–thymic primordium. Therefore, further approaches, including the precise regulation of transcription factors such as GCM2, the application of three-dimensional organoid cultures, and the reconstruction of a supportive developmental niche, will be required.

## 5. Conclusions

In conclusion, the human iPSC-based in vitro differentiation protocols used in the present study induce parathyroid–thymic primordium differentiation by activating pharyngeal pouch development-related genes. However, these protocols are limited in their ability to efficiently generate mature physiological parathyroid cells, thereby highlighting a developmental bottleneck at the transition from primordium to parathyroid lineage commitment.

## Data Availability

The original contributions presented in this study are included in the article/Supplementary Materials. Further inquiries can be directed to the corresponding author.

## Supplementary Materials

The following supporting information can be downloaded at: https://www.mdpi.com/article/10.3390/cells15141252/s1, Figure S1: The expression and localization of EYA1 and PTH in the iPSC-derived parathyroid lineage–differentiated cells; Figure S2: Parathyroid lineage differentiation in 3D spheroid culture; Table S1: Primers used for quantitative real-time PCR in this study.
