# Supplementary material for: A Transcriptomic Analysis of Human iPSC-Derived Parathyroid Lineage Cells Reveals Limited Maturation Beyond the Parathyroid–Thymic Primordium
Source: Cells. 2026 Jul 11;15(14):1252. doi: 10.3390/cells15141252 (PMC13406470; doi:10.3390/cells15141252)
Supplement: Supplementary file 1 [file cells-15-01252-s001.zip › cells-4299744-supplementary.pdf]

Supplementary information

Supplementary Materials

Three-dimensional spheroid culture for parathyroid differentiation

For differentiation in 3D spheroid culture, human iPSCs were dissociated with accutase (Nacalai Tesque) and re-plated onto 96 well round bottom plate (Corning) at a density of 50,000 cells/cm2. On the next day, the cells were cultured in RPMI 1640 (Nacalai Tesque) supplemented with 2% B-27 supplement (Thermo Fisher Scien-tific) containing 3 μM CHIR-99021 (Focus Biomolecules) and 100 ng/mL activin A (API Co., Ltd.). The cells were continuously aggregated within differentiation medium (RPMI 1640 supplemented with 2% B-27 supplement) containing 100 ng/mL activin A and 500 nM LDN 193189 (Merck) for another 2 days. The cells were then cultured with differentiation medium for three days. Spheroid-forming definitive endoderm were then re-plated into low adhesion cell cultureware (Ultra-low attachment 6-well plates; Corning) and cultured with 1 μM ATRA (LKT Labora-tories) and 2.5 μM IWR1-endo (Fujifilm) for 4 days. Finally, in stage 3, we treated the spheroids with 100 ng/mL SHH (PeproTech) and 50 ng/mL activin A.

Total RNA preparation and quantitative real-time PCR

After 15 days and 23 days (2D culture) or 10days and 24days (3D culture) of differentiation induction, total RNA was isolated using the RNeasy Plus Mini kit (Qiagen). Then, complementary DNA was synthesized using the High-Capacity cDNA Reverse Transcription Kit (Thermo Fisher Scientific), and quantitative real-time PCR was performed with the THUNDERBIRD Next SYBR qPCR Mix (TOYOBO Co., Ltd., Tokyo, Japan) on a real-time PCR cycler (StepOnePlus; Thermo Fisher Scien-tific). Real-time RT-PCR amplification was conducted with a program of 95 °C for 30 seconds, 40 cycles of 95 °C for 5 seconds, 60 °C for 30 seconds. The primer sets used in the real-time PCR reactions are listed in **Table S1**.

Figure S1

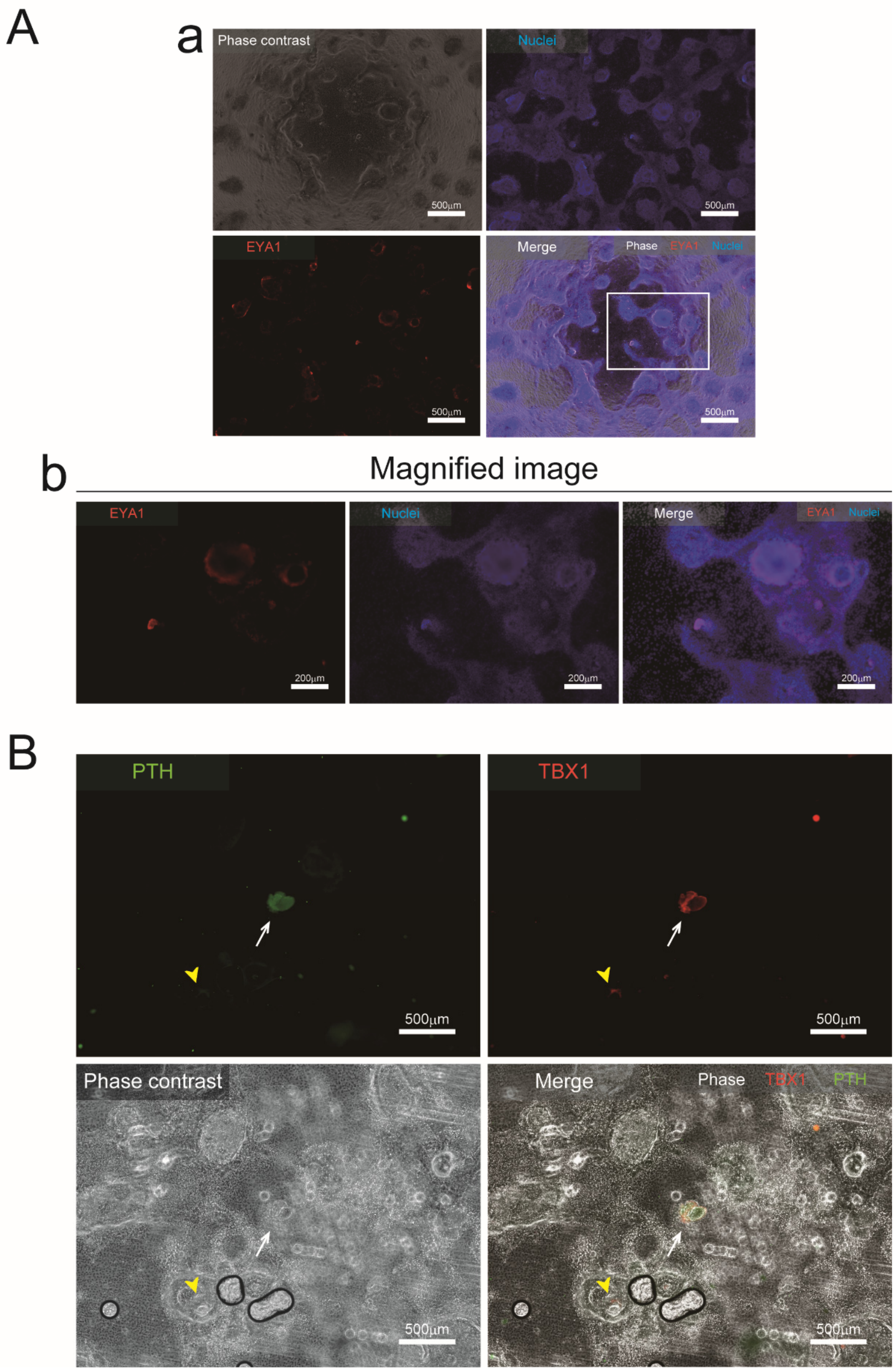
A
a
Phase contrast
Nuclei
EYA1
Merge
Phase EYA1 Nuclei
500μm
b
Magnified image
EYA1
Nuclei
Merge
EYA1 Nuclei
200μm
B
PTH
TBX1
Phase contrast
Merge
Phase TBX1 PTH
500μm

Figure S1. The expression and localization of EYA1 and PTH in the iPSC-derived parathyroid lineage–differentiated cells.

(A) Representative phase contrast image and fluorescence images of differentiated cells in culture. The fluorescence images of EYA1 (red) and nuclei (blue) in parathyroid differentiated cells are shown. Low **(a**, scale bars: 500 μm**)** and high **(b**, scale bars: 200 μm**)** magnified images of parathyroid differentiated cells are shown individually. The area indicated by a white square in low-magnification image is magnified. **(B)** Representative fluorescence images of PTH (green) and TBX1 (red) in parathyroid differentiated cells are shown (scale bars: 500 μm). Arrows indicate cell clusters co-expressing PTH and TBX1, whereas yellow arrowheads indicate cell clusters expressing TBX1 alone.

Figure S2

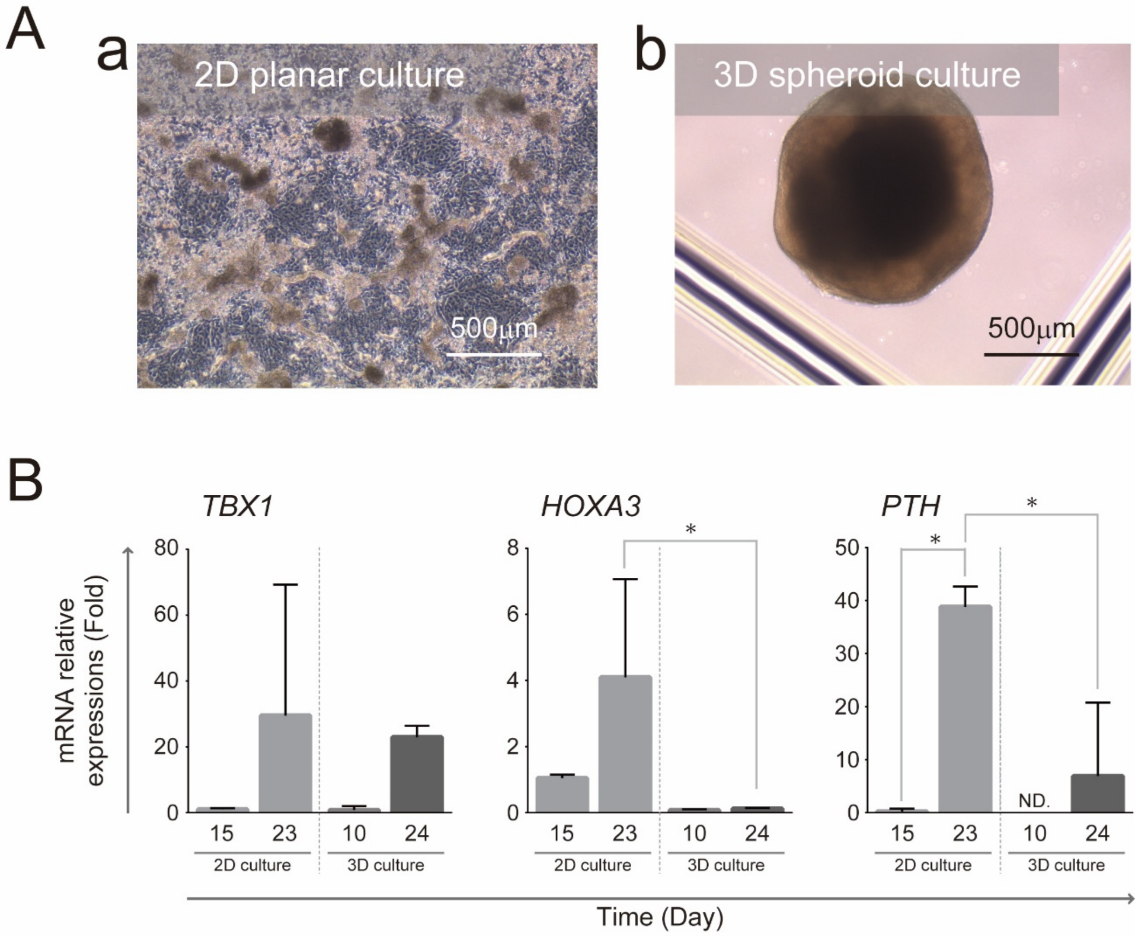

Figure S2. Parathyroid lineage differentiation in 3D spheroid culture.

(A) Representative phase contrast images of parathyroid lineage–induced cells cultured under **(a)** 2D planar (day 23) and **(b)** 3D spheroid (day 24) conditions (scale bars: 500 μm). **(B)** The gene expression of parathyroid lineage-induced human iPSCs in 2D planar culture and 3D spheroid culture was analyzed by quantitative real-time PCR. Gene expression levels were normalized to *GAPDH*. The relative expression levels of *TBX1*, *HOXA3* and *PTH* were compared between 2D planar culture at day 15 and 23, and 3D spheroid culture at day 10 and 24. The bars represent the mean ± S.D. of four independent measurements. These expressions were evaluated by a one-way ANOVA with Tukey's multiple comparison procedure and statistical significance was defined as follows: * $P < 0.05$; ND., not detected.

Table S1. Primers used for quantitative real-time PCR in this study

Nucleotide sequences for real-time PCR		
**Amplicon**	**Nucleotide sequence**	
GAPDH	Forward	5'- GCACCGTCAAGGCTGAGAAC -3'
	Reverse	5'- TGGTGAAGACGCCAGTGGA -3'
HOXA3	Forward	5'- TCAAAGGCGCTTTGAAACAG -3'
	Reverse	5'- GACTGTTCACCAGCATACACACAC -3'
PTH	Forward	5'- CAGAGAATTGGGAGTGACATCATCT -3'
	Reverse	5'- TGCATAAGCTGTATTTCACTCACAG -3'
TBX1	Forward	5'- ATGTGGACCCACGCAAAGATAG -3'
	Reverse	5'- GGCAATCTTGAGCTGCGTGA -3'
